# Comprehensive Quantitative Analysis on Privacy Leak Behavior

**DOI:** 10.1371/journal.pone.0073410

**Published:** 2013-09-16

**Authors:** Lejun Fan, Yuanzhuo Wang, Xiaolong Jin, Jingyuan Li, Xueqi Cheng, Shuyuan Jin

**Affiliations:** Institute of Computing Technology, Chinese Academy of Sciences, Beijing, China; University of Catania, Italy

## Abstract

Privacy information is prone to be leaked by illegal software providers with various motivations. Privacy leak behavior has thus become an important research issue of cyber security. However, existing approaches can only qualitatively analyze privacy leak behavior of software applications. No quantitative approach, to the best of our knowledge, has been developed in the open literature. To fill this gap, in this paper we propose for the first time four quantitative metrics, namely, *possibility*, *severity*, *crypticity*, and *manipulability*, for privacy leak behavior analysis based on Privacy Petri Net (PPN). In order to compare the privacy leak behavior among different software, we further propose a comprehensive metric, namely, *overall leak degree*, based on these four metrics. Finally, we validate the effectiveness of the proposed approach using real-world software applications. The experimental results demonstrate that our approach can quantitatively analyze the privacy leak behaviors of various software types and reveal their characteristics from different aspects.

## Introduction

Privacy leak behavior invading users' data privacy has been widely discovered in different types of software and has thus become a very important research issue of cyber security. Existing approaches to analyzing privacy leak behavior can be classified into two categories: black-box approaches and white-box approaches. The black-box approaches focus on the input data and output network traffic of software, which rapidly find privacy data with well-defined format (e.g., credit card number) by evaluating the variation of privacy information between input and output data [1]–[2]. However, these approaches face the limitation of packet obfuscating techniques (e.g., encrypted connections, message reordering and traffic randomization) [1]. In comparison to the black-box approaches, the white-box approaches can accurately analyze privacy leak behavior in detail [3]. These approaches can be further divided into static analysis approaches and dynamic analysis approaches. Static analysis approaches reveal the accurate data flow from binary executable files of the target software [4], which also confront code obfuscating problems (e.g., code morphing, packer and opaque constant) [5]. Dynamic analysis approaches, which detect the runtime data flow by tracing the execution of the target software, are widely used in software behavior analysis [6]–[7]. Unfortunately, dynamic analysis approaches have their shortages in solving problems such as multiple paths [8] and dormant functionality [9]. Although the above black-box and white-box approaches have been proposed for years, privacy leak behavior analysis still suffers from two common problems. First, there are no quantitative evaluation metrics for analyzing privacy leak behavior. Second, there is no metric for comprehensively comparing the overall degree of privacy leak of different software applications. Such a metric is very important, because it can indicate the overall threat level of the tested software application.

To overcome these two problems, we propose, for the first time, a set of desired quantitative metrics based on an abstract model called Privacy Petri Net (PPN) presented in [10], which characterizes the entire privacy leak procedure with more high-level description. Specifically, we propose and define four quantitative metrics, i.e., *possibility*, *severity*, *crypticity*, and *manipulability*, to characterize different aspects of privacy leak behavior and make the analysis more understandable. In order to compare the privacy leak behavior of different software applications, we further present a comprehensive metric, i.e., the overall privacy leak degree, by virtue of the above four metrics. Finally, we apply the proposed approach to real-world software applications and show that it can quantitatively analyze the privacy leak behavior of various software types and find their characteristics from different aspects.

## Model

Privacy Petri Net (PPN) is a high-level Petri net dedicated to privacy leak behavior analysis [10], which has three main features. Firstly, PPN has formal mathematical definitions of syntax and semantics, which provide a precise specification on the target software behavior, so as to essentially define various behavior properties. Secondly, PPN has powerful modeling primitives of graphical abstraction. Specific graph structures can be used to identify unique private information leak behavior. Finally, PPN is modularized and can thus be used to build hierarchical models. By virtue of these features, we can use PPN to model different types of privacy leak behaviors and construct more complicated and powerful models. Here we first make a brief introduction to PPN and then present seven typical modules of PPN.

### Definition of Privacy Petri Net (PPN)

**Figure 1:**
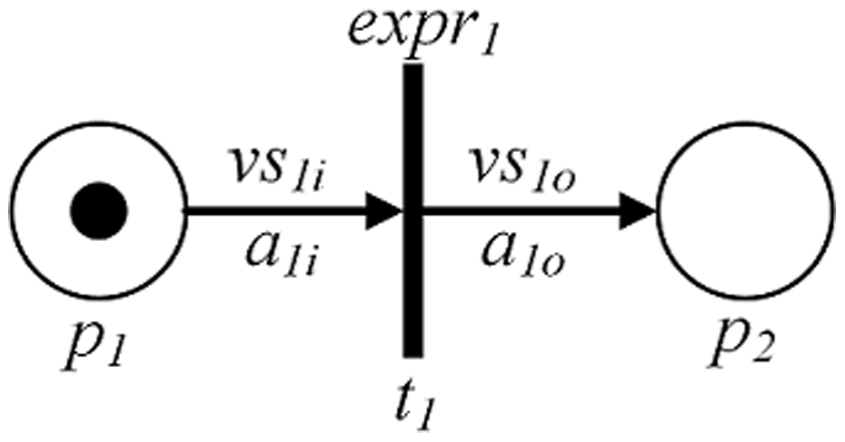
A schematic diagram of a PPN.

Fig. 1 presents a schematic diagram of a PPN that can be formally defined as follows.

**Figure 2:**
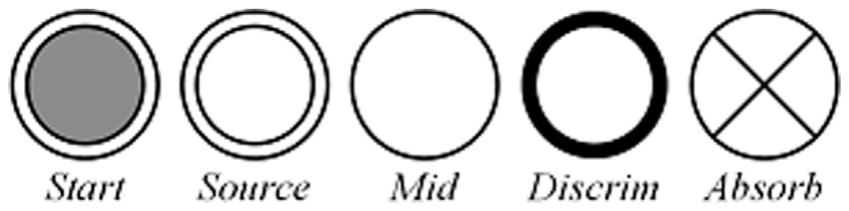
Position icons.

**Definition 1.** A Privacy Petri Net (PPN) is a seven-tuple, PPN = (*P*, *T*, *A*, *INST*, *f_pos_*, *f_tran_*, *f_arc_*), where:

1. *P* is a finite set of positions. Each position in *P* denotes a local status of a subroutine of software execution.
2. *T* is a finite set of transitions. Each transition in *T* denotes a system call or an API call.
3. *A*⊆{*P*×*T*} ∪ {*T*×*P*} is a set of directed arcs that connect positions and transitions. The arcs heading to and emiting from a transition are called input and output arcs of the transition, respectively (e.g., *a_1i_* and *a_1o_* in Fig. 1).
4. *INST* = {(*Ctg*, *Cont*, *Proc*)} is the set of instances of privacy data sources in each access. Each instance in *INST* can be denoted by a token with three special attributes, *Ctg*, *Cont*, and *Proc*. *Ctg* is the category of data sources, taking possible values from the set, {“*File*”, “*Application*”, “*System*”, “*Dynamic*”}. *Cont* is the content of privacy data which differs according to its category. For example, for a file, *Cont* is its name or path, while for a system, *Cont* is the registry key name and key value. *Proc* denotes the steps/path that the token traverses through the whole PPN, which is a sequence of positions and transitions, denoted as *Proc* = *p_1_t_1_p_2_*…*p_n−1_t_n−1_p_n_* (*n*≥1), where *p_i_*∈*P* (*i* = 1, 2, …, *n*) and *t_j_*∈*T* (*j* = 1, 2, …, *n*−1). These attributes depict the fundamental information of the privacy leakage, which are initialized with an empty value when the token is created.
5. *f_pos_* is a mapping *f_pos_ : P→{“Start”,“Source”,“Absorb”,“Mid”,“Discrim”}*. It assigns a property to each position to indicate its role in privacy leak. In a PPN, different positions are indicated with distinct icons as depicted in Fig. 2. A “*Start*” position spawns new tokens with unassigned privacy attributes. A “*Source*” position denotes the access point of a privacy data source. A “*Discrim*” position means that the privacy leak behavior can be discriminated when a token reaches it. An “*Absorb*” position implies that the operation on the related data source has been checked and is not considered as privacy leak behavior. “*Mid*” positions indicate all other positions. In order to implement the modularization of PPNs, a few *PPN module* will be defined later for the privacy leak procedure. Each PPN module must have at least one “*start*” position, one “*absorb*” position, one “*source*” position and one “*discrim*” position to depict a complete privacy leak procedure.
6. *f_arc_* is an arc function set that assigns a set of variables to each arc and is denoted by *f_arc_* : *A*→*VARSET*. The variable set assigned to an input arc is called an *input variable set* (e.g., *vs_1i_* in Fig. 1), while the one assigned to an output arc is called an *output variable set* (e.g., *vs_1o_* in Fig. 1). All variables can be categorized into two main kinds. One is the parameters used to support the system or API calls, such as, integer, float, handle, pointer, string and struct. The other is used to support the privacy attributes. They are mainly calculated from the parameter variables.
7. *f_tran_* is a transition function that maps an expression to each transition and is denoted by *f_tran_*: *T*→*EXPR*. *EXPR* is a Boolean operation of some conditions on checking privacy leak, which intrigue the corresponding transition. These conditions include many categories, such as, the name checking of the current system call or API call, the value checking of the current system environment variable, globe system configuration checking, and other predefined constraint conditions. There must be at least one condition checking the name of the current system call or API call, because we characterize the software behavior mainly by the call sequence.

Next, we present two concepts and an important theorem about PPN for checking the privacy leak behavior.

**Definiton 2.** Behavior path set

Let *C* = *c_1_c_2_…c_n_* (*n*≥1) be a call sequence of the target software application and *inst_1_*, *inst_2_*, …, *inst_m_* (*m≥1*) be privacy instances spawned when the calls in *C* are checked to trigger the transitions. The behavior path set, *BPS*, is the set of move traces of all privacy instances, denoted as *BPS* = {*inst_1_*.*proc*, *inst_2_*.*proc*, …, *inst_m_*.*proc*}. Each call sequence *C* can be mapped to a unique *BPS* to describe the privacy leak related behavior of the target software application.

**Definiton 3.** Leak path and leak reachability

A *leak path* is a special move trace of a privacy instance in *BPS*, where there must exist a source position and a discrimination position. Let *inst_k_*.*proc = p_1_t_1_p_2_*…*p_n−1_t_n−1_p_n_* and *inst_k_*.*proc*∈*BPS*. We have: *inst_k_.proc* is a leak path ⇔ the privacy instance *inst_k_* has leak reachability⇔∃*i*, *j* : *f_pos_*(*p_i_*) = “*source*”∧*f_pos_*(*p_j_*) = “*discrim*”.

With the leak reachability property we can verify whether a certain type of private information contained in the target software application is leaked as presented in Theorem 1.

**Theorem 1.** (Discrimination Theorem) *If a privacy instance, inst, spawned in a PPN module, m, has leak reachability, then the call sequence C of the target software application contains a privacy leak behavior procedure that modeled by the PPN module.*

The proof of Theorem 1 can be found in our previous work [10].

### Typical Modules of PPN

**Figure 3:**
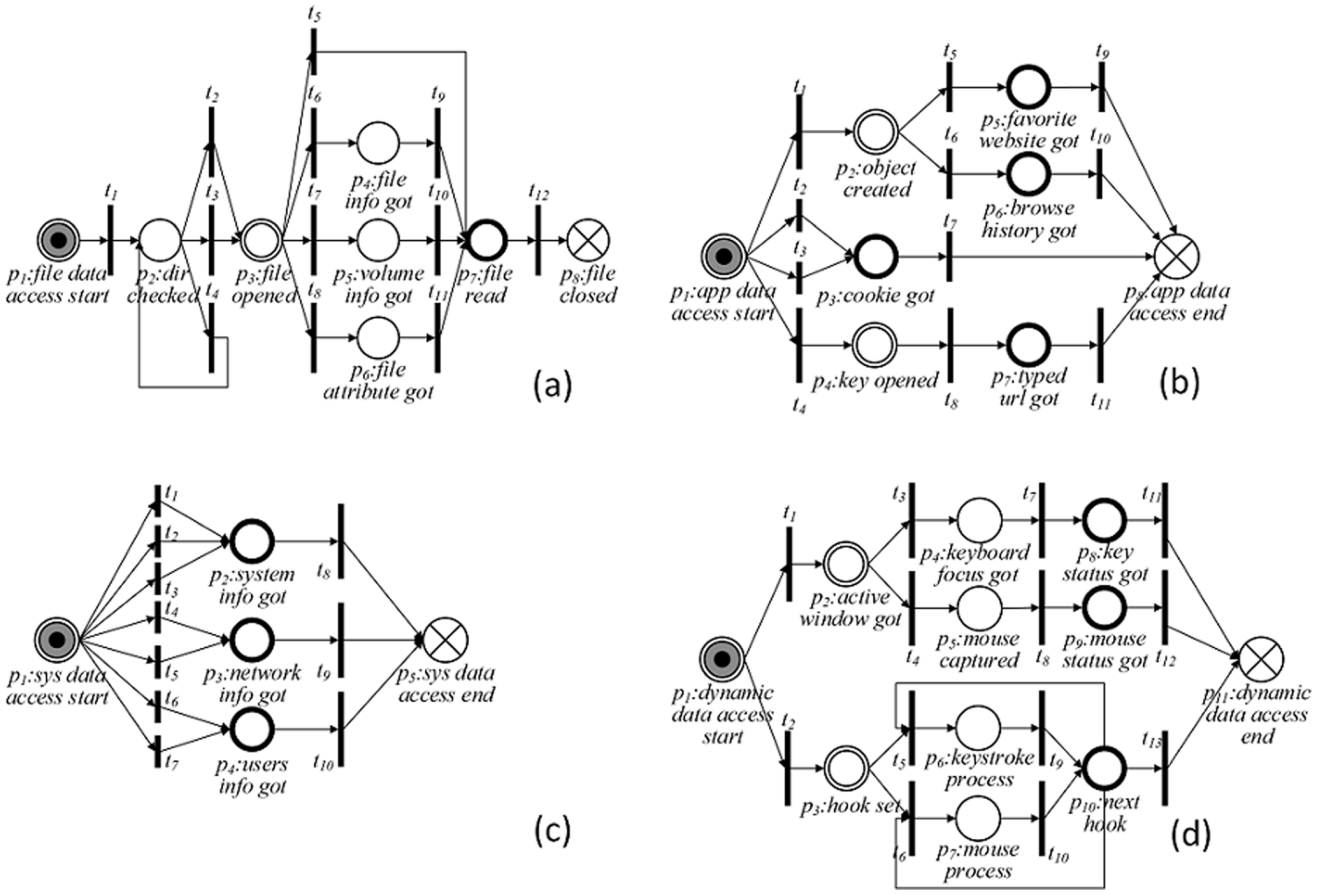
Typical module for privacy data accessing. (a) Module *m_nfda_* : PPN module for ordinary file data access; (b)Module *m_ada_* : PPN module for application data access; (c) Module *m_sda_* : PPN module for system data access; (d) Module *m_dda_* : PPN module for dynamic data access.

**Figure 4:**
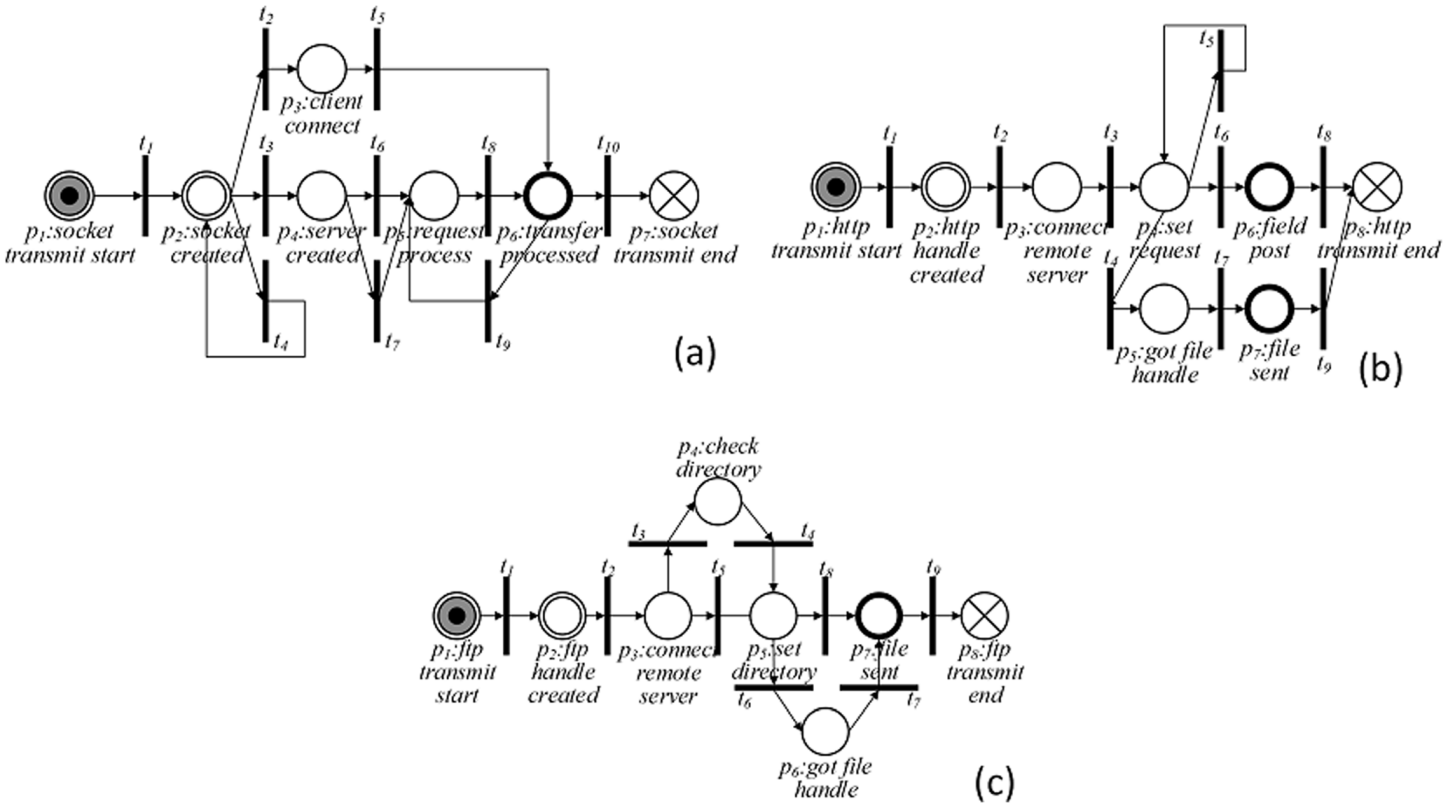
Typical module for privacy data transmission. (a)Module *m_socket_* : PPN module for socket connection; (b)Module *m_http_* : PPN module for HTTP connection; (c) Module *m_ftp_* : PPN module for FTP connection.

In PPN, there are a few typical PPN modules corresponding to sub-procedures of privacy leak behavior of Windows software. Specifically, there are four typical PPN modules corresponding to unauthorized data access (see Fig. 3) and three PPN modules for covert network transmission (see Fig. 4). The integration of some typical PPN modules may generate more complicated PPNs.

The PPN modules presented in Fig. 3 (a)–(d) correspond to the behavior of software for accessing ordinary files, application data, system data and dynamic data, respectively. It can be seen from Fig. 3(a) that to access data in an ordinary file: (1) The target software should check the possible directories to find the file by “NtQueryDirectoryFile” or “NtNotifyChangeDirectoryFile”, which can be found in Table 1. Then the corresponding transitions *t_1_* or *t_4_* are triggered and the token moves to position *p_2_*; (2) The software application acquires the file handle by “NtCreateFile” or “NtOpenFile”. Then the corresponding transitions *t_2_* or *t_3_* are triggered and the token moves to position *p_3_*; (3) Finally, the application obtains the properties or content of the file by “NtFsControlFile”. Then the corresponding transition *t_5_* is triggered and the token moves to position *p_7_*. There are also three other possible leak paths from position *p_3_* to position *p_7_*: *p_3_t_6_p_4_t_9_p_7_*, *p_3_t_7_p_5_t_10_p_7_* and *p_3_t_8_p_6_t_11_p_7_*. To differentiate from the legitimate file access, we have to check the file path for further verification. For example in Table 1, the “Path Check” in the *EXPR* of *t_2_* and *t_3_*, only software installation path, system path and pathes created by software are permitted to be accessed. For the sake of space limitation, we do not describe the other three modules in Fig. 3 in detail.

**Table 1:** Details Of The Module Presented In Fig. 3(A).

	*EXPR*	*Input VarSet*	*Output VarSet*
*t_1_*	“NtQueryDirectoryFile”	Dir name	“Ctg”
*t_2_*	“NtCreateFile”∧Path Check	File name	File handle
*t_3_*	“NtOpenFile”∧ Path Check	File name	File handle
*t_4_*	“NtNotifyChangeDirectoryFile”	Dir name	File handle
*t_5_*	“NtFsControlFile”	Ctl Code	Pointer,“Cont”
*t_6_*, *t_7_*, *t_8_*	“NtQueryInformationFile” “NtQueryAttributeFile” “NtQueryVolumeInformationFile	File handle	Pointer,“Cont”
*t_9_*,*t_10_*,*t_11_*	“NtReadFile”	File handle	Pointer,“Cont”
*t_12_*	“NtClose”	File handle	NULL

The PPN modules presented in Fig. 4 (a)–(c) for coverting network transmission correspond to three kinds of connections, namely, socket connection, FTP connection, and HTTP connection, respectively. In Fig. 4(a), it can be observed that to build a socket connection, the target software application should first create a socket and further build the server side or the client side. The application can then send privacy data and receive remote data. In Fig. 4(b), for building an HTTP connection to leak privacy data, the target software application has to first create an Internet handle and set up a connection to a remote HTTP server. Next, it creates an HTTP request and adds HTTP headers. Finally, it sends data by posting an HTTP request. As depicted in Fig. 4(c), an FTP connection is used to transfer privacy data in files. It is especially suitable for a large number of files or a single large file. The leak procedure is similar to that of an HTTP connection: An Internet handle and connection are created first. Then, the work directory for file transmission is set. After these two steps, privacy data is leaked.

## Methods

*Privacy leak* can mainly be described from four aspects, namely, content, source, procedure and destination. Leak content refers to which kind of privacy data is leaked. Leak source means the storage form of privacy data. Leak procedure records the related system call sequence and the final destination of privacy data. Finally, leak destination is the remote server to which the privacy data is sent. In this paper, we propose to analyze privacy leak behavior from the above four aspects using PPN. We will not only qualitatively but also quantitatively analyze privacy leak behavior by proposing four metrics, i.e., *possibility*, *severity*, *manipulability*, and *crypticity*. Possibility is a basic metric denoting the probability that privacy leak behavior may occur. Severity denotes the negative consequence of an actual privacy leak behavior. Manipulability implies the tendency that the software application may be used by, or cooperated with, other applications to leak privacy data. Crypticity indicates the difficulty to detect the corresponding privacy leak behavior. These quantitative metrics characterize different aspects of privacy leak behavior, the combination of which makes the analysis more understandable and comprehensive. They are calculated mainly based on the output of PPN, i.e., the behavior path set (*BPS*). As presented in Definition 3, software behavior can be mapped to a set of traces of privacy instances moving across positions and transitions in the PPN corresponding to the execution of the target software application.

### Weights of Transitions and PPN Modules

For a behavior path *σ_pathi_* = *inst_i_.proc = p_i1_t_i1_p_i2_*…*p_i(n−1)_t_i(n−1)_p_in_,* we first assign a weight to each transition *t_j_* (1≤*j*≤*n*−1) according to its importance in the privacy leak procedure. A high weight means that the corresponding transition is more likely to be triggered in existing malware and is more important to privacy leak behavior. The weights are actually the statistical probabilities of these transitions. Specifically, given a set of software applications, they are run for a long enough period of time such that no more type of transitions can be observed. Assume that there are totally *N* types of transitions. In this situation, let 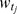 (0≤*w_tj_*≤1, 1≤*j*≤*N*) be the weight of transition *t_j_*. If the corresponding system/API call is invoked, its call counter *c_j_* increases by 1. Consequently, 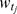 can be calculated by
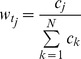
(1)Additionally, we divide the entire behavior path into different parts based on the typical PPN modules. Assume that a behavior path 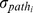 passes through *m* modules. 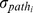 can then be denoted as 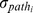 = *σ_1_σ_2_…σ_m_*, where *σ_i_* = *p_i1_t_i1_p_i2_*…*p_i(n−1)_t_i(n−1)_p_in_* (1≤*i*≤*m*). We also assign a weight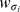 to the path fragment *σ_i_* according to the negative consequence of the corresponding PPN module. Such a negative consequence is affected by many factors, including the content, source and final destination of the leaked privacy data. Furthermore, the application scenario of the software application also influences the severity of the negative consequence. For instance, for a business server, the leak of the system data may expose the vulnerability of the system and make the server more vulnerable to network intrusion. For a personal computer, the leak of some dynamic data (e.g., bank account information and password) may cause financial loss. Considering all these factors, we sort the typical PPN modules in an ascending order based on the severity of their negative consequences and assign the module weight 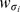 accordingly. More specifically, in this paper the seven PPN modules are sorted as *m_sda_*, *m_nfda_*, *m_ada_*, *m_dda_*, *m_http_*, *m_socket_*, *m_ftp_*. Therefore, since 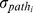 contains *m* predefined PPN modules, their weights 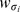 are assigned values from *1* to *m* in an ascending order. These weights will be used to calculate the severity and manipulability of privacy leak behavior.

### Calculation of Quantitative Metrics

In this subsection, we show how to calculate the four metrics based on the aforementioned behavior paths.

- Possibility

Possibility implies the probability that the paths in the BPS actually form leak paths. It is the fundamental metric for evaluating privacy leak behavior. Assume that *σ_path_* is a behavior path in the BPS that contains *n_j_* transitions and *n_j_*+1 positions. Then, the possibility *p*(*σ_pathj_*) can be calculated as: 
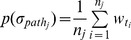
. If there are *N* behavior paths in the *BPS*, the corresponding possibility can be obtained by:
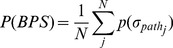
(2)

- Severity

The calculation of severity is also based on the leak paths in the *BPS*. Assume that leak path 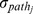 contains *m_j_* PPN modules. Its severity *s*(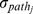) can be calculated as 
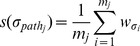
. Further, the overall severity of the application under test can be denoted as:
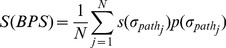
(3)where *N* is the numbers of leak paths in the BPS. Severity is a comprehensive and intuitive metric for evaluating privacy leak behavior. The higher the severity, the severer the loss caused by the leak behavior.

- Manipulability

Manipulability concerns whether the software application can be manipulated by other applications. Although some application only performs sub-procedure of a privacy leak behavior and thus has low possibility and severity, they may become a component of other malicious software in the multi-process collaborative work mode. Typically, manipulability has two modes, the relay race mode and the master/slave mode. The former splits its main functionality flow into two or more consequent parts, while the latter uses a master process to create and control slave processes. A feature they both have is that they split the leak behavior into several partial procedures. Therefore, manipulability should be evaluated for the leak behavior that is only partially completed. Specifically, it can be calculated for the paths that are not leak paths but still contain a source position or a discrimination position. And such paths should also be weighted by their severity. In general, manipulability can be calculated as
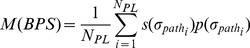
(4)where *N_PL_* is the number of partial leak paths in the *BPS*. The above definition of manipulability simplifies the metric in practice, but still captures the core of manipulation techniques. Although high manipulability does not lead to direct damage to privacy data, it is still necessary to pay special attention to it.

- Crypticity

Crypticity is to evaluate the ability of privacy leak behavior to avoiding detection. Dedicately designed software applications always employ hiding and obfuscating techniques to improve their resistance against anti-virus software. There are many hiding and obfuscating techniques that privacy leak software applications often use. Most of them employ additional operations to disguise obvious malicious behavior. Assume that within the system call sequence of the target software application, *n_ei_* extra system calls are traced but not represented by transitions between the start position and the end position of a behavior path 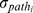. The crypticity can then be calculated by
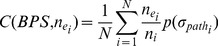
(5)


Equation (5) indicates that the more the obfuscating or hiding operations for disguising the privacy leak behavior, the higher crypticity. Crypticity is essentially a complementary metric to severity. The software application with high severity may be easily detected and removed by anti-virus software, although high crypticity may help the privacy leak software application be active for a longer time and thus amplify its damage significantly.

**Figure 5:**
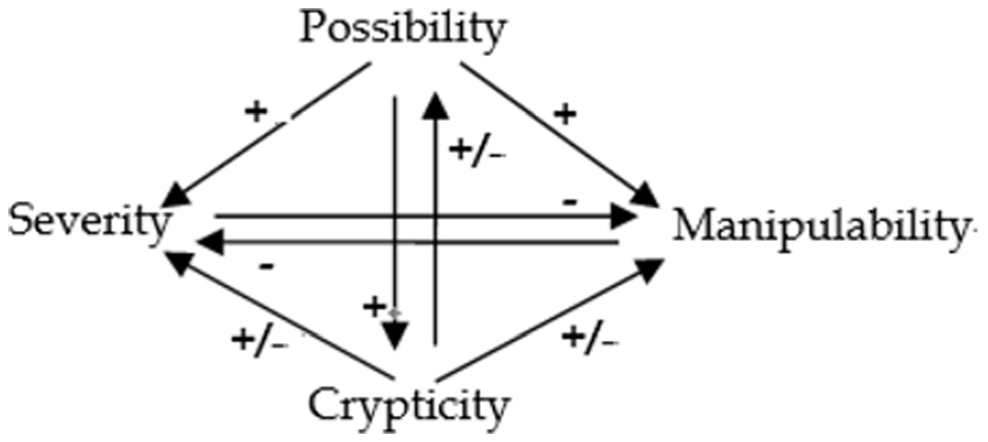
Interrelationship among the four metrics.

Although the four metrics describe privacy leak behavior from different aspects, they have interrelations between each other. Fig. 5 summarizes the interrelationship, where the plus and minus signs indicate the positive and negative correlation between two metrics, respectively. The “+/−” signs denote the uncertain but potential interrelations. We can see from Fig. 5 that the four metrics constitute a closely linked entirety.

Possibility is a fundamental metric for evaluating privacy leak behavior. High possibility means a large probability 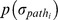 that the behavior path exists. Because only if a privacy leak behavior probably occurs, severity, manipulability and crypticity make sense. Therefore, possibility is a prerequisite of severity, manipulability and crypticity and 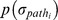 thus appears in their formulae as a factor. Severity and manipulability are complementary to each other, which characterize the straightforward and potential negative consequence of the privacy leak behavior of the software application under consideration, respectively. Crypticity is more special than other metrics. It may have an indirect impact on other metrics, because the obfuscating technique can conceal extra malicious operations. For example, it may cause unknown negative consequences, and thus increase severity and manipulability. Therefore, even if the other metrics are trivial, a software application with high cypticity is still likely to exhibit privacy leak behavior.

### The Overall Leak Degree

**Figure 6:**
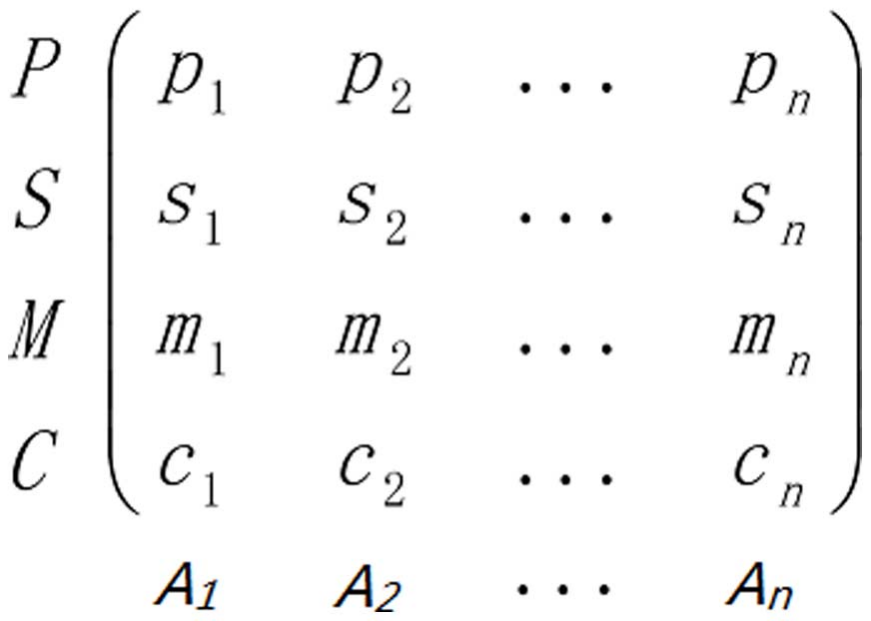
Privacy leak metric matrix.

In order to compare the overall degree of privacy leak behavior of different software applications, a comprehensive metric need to be proposed based on the possibility, severity, manipulability and crypticity metrics. Quantifying the overall degree of privacy leak behavior can be abstracted as a Multiple Attribute Decision Making (MADM) problem. An MADM problem aims to choose an item from a set of alternatives characterized by multiple attributes. Consider an MADM problem with *n* alternatives that denote the values of the four metrics of *n* software applications under test. A matrix corresponding to privacy leak metric is shown in Fig. 6, where the rows, denoted as *P, S, M, C*, correspond to the four metrics, and the columes, denoted as *A_1_*, *A_2_*, …, *A_n_*, correspond to *n* alternative, i.e., the *n* software applications, respectively.

As aforementioned, the four metrics are interrelated. Therefore, in order to evaluate the overall degree of privacy leak behavior of the software application under test, we propose an algorithm, called PLEAS (Privacy Leak ovErall quAntitative analysiS), based on the Principal Component Analysis (PCA) to solve the above MADM problem. PCA is a method for identifying patterns in interrelated data and expressing the data in a way that highlights their similarities and differences. PCA can guarantee that PLEAS is able to obtain the most meaningful overall degree based on four metrics for comparative analysis between different software applications. With PLEAS, the alternatives *A_1_*, *A_2_*, …, *A_n_* will be finally ranked. The higher the ranking value is, the more severe the privacy leak behavior of the corresponding software application will be. Readers are referred to Supporting Information S1 for more details about PLEAS.

## Results

This section validates the proposed PPN based approach to quantitative analysis on privacy leak behavior. Specifically, we first present an overview of the analysis environment and software set used for experiments. Next, we show a case study on an Internet software tool in order to demonstrate the effectiveness of the developed approach. Finally, as a direct application, we adopt the approach to carry out a comparative analysis on different software categories and discuss the experimental results.

### Analysis Environment

In the experiments, VMware Workstation 7.0 is used as a virtual machine platform to build a Windows OS image. The core algorithm of the PPN model is implemented by Python 3.1. API monitor2 r9 is adopted to extract runtime execution traces of the set of software tools under test. All the software tools are installed and tested in different snapshots of the virtual host to avoid mutual interference.

We selected the programs contained in the Windows OS (WIN 7 Professional Edition) as the evaluation baseline environment. This program set includes system utilities under the system path “C:\\Windows\\System32” and the preinstalled software applications under the program path “C:\\Program Files”. For some software tools that need user interaction, we tested them manually for 10 minutes. We then calculated the quantitative metrics of these tools as the baseline values of the baseline environment. After averaging the results of 1,026 benign software tools, the baseline values of the total leak paths, possibility, severity, manipulability and crypticity are 5, 0.35, 2.78, 2.59, and 3.26, respectively.

For the purpose of experiments, we also downloaded seven main categories of software tools from the well-known free software download website, Download.com: (1) entertainment software, including games, music software, video software, etc; (2) operating systems utilities, including file management software, backup software, automation software, desktop enhancement, etc; (3) Internet software, including browsers, email clients, file download/upload software, social networking software, etc; (4) business software, including office suites, account and billing software, E-commerce software, etc; (5) educational software, including E-book software, language software, student tools, science software, etc; (6) developer tools, including IDEs, debugging software, coding utility; (7) security software, including anti-virus software, encryption software, firewalls, etc. Specially, for each category, we downloaded the top ten software applications according to their popularity.

### Case Study on a Web Browser

**Figure 7:**
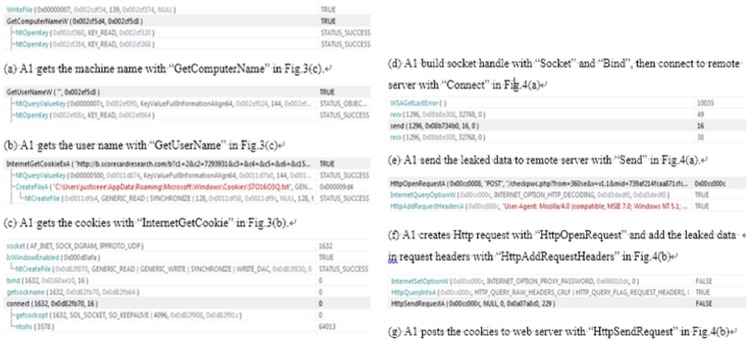
The call sequence fragment of A1.

**Figure 8:**
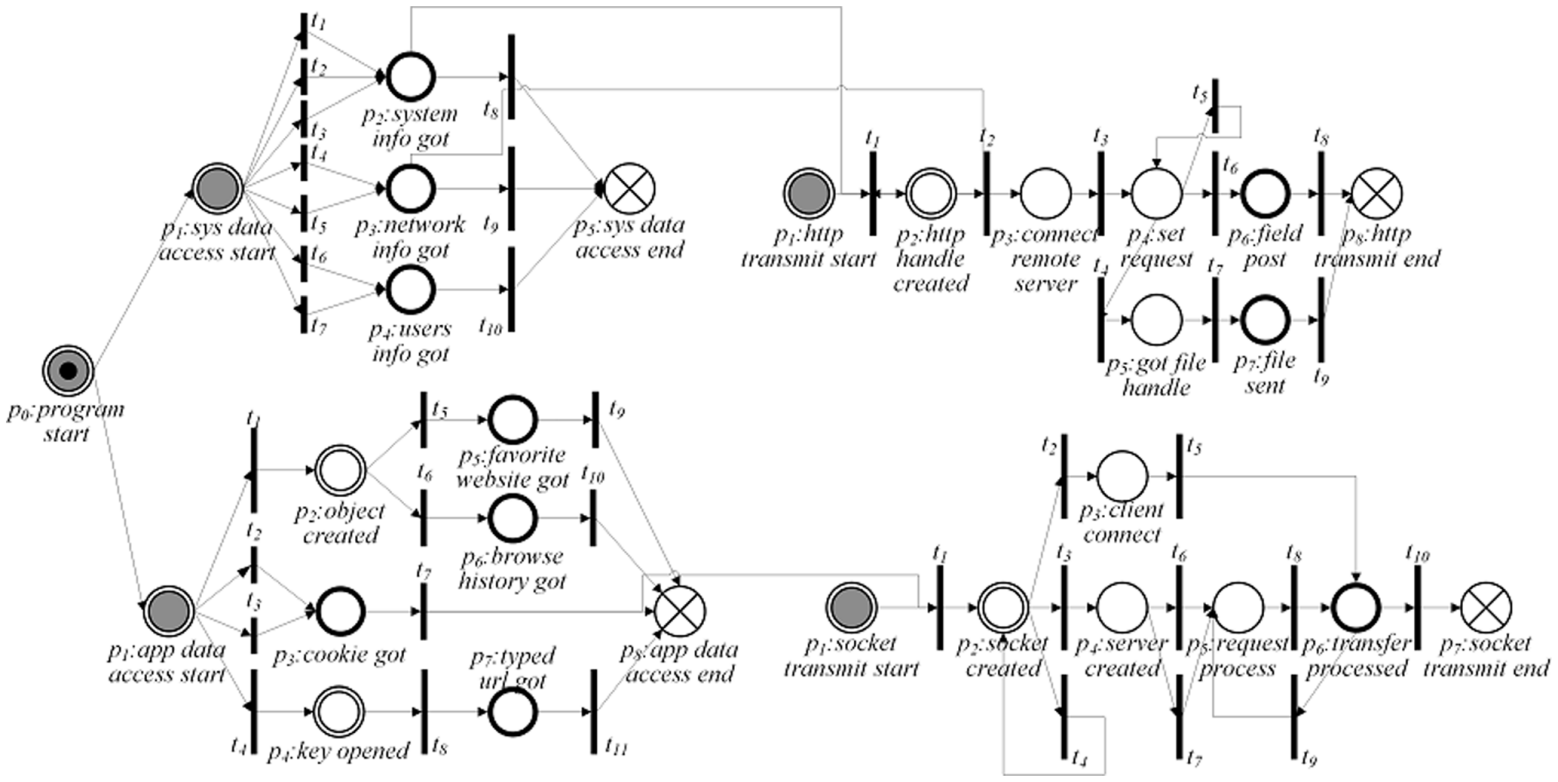
The PPN presenting the privacy leak behavior of A1.

A web browser, referred to as A1 in this paper, has many plug-in enabled functions. Its call sequence fragments are depicted in Fig. 7. If the user installs a plug-in for managing the browse history and the favorite folder, it exposes to leak behavior, such as, collecting the cookies of some websitses and the sensitive system info beyond its authority. According to the call sequence, “GetComputerName, GetUserName, InternetGetCookie, Socket, Bind, Connect, Send”, we obtained the PPN model as shown in Fig. 8. By tracing the execution of A1, we collected 15 leak behavior paths within 121 behavior paths. The number of leak behavior paths of A1 is larger than that of the baseline environment. The possibility, *P*, of the leak paths of A1 is 0.67, which is much higher than the corresponding baseline value, 0.35, indicating that A1 very probably has privacy leak behavior. According to the characteristic of web browsers, system data and application data that contain the browse history, usage habit and favorite websites of users are prone to be leaked. Therefore, the severity *S* of A1 is obtained to be 4.15. Due to the high percentage of the partial behavior paths, although A1 does not have many complete leak paths, its manipulability *M* was 3.39, higher than the baseline value. In addition, since a few obfuscating techniques are adopted by A1, its crypticity, *P*, is 9.51. The above results show that A1 more probably leaks privacy data, causes more severe consequence, and is more probably manipulated by other software tools, than the baseline program set in WIN 7, which is basically a reasonable observation.

**Figure 9:**
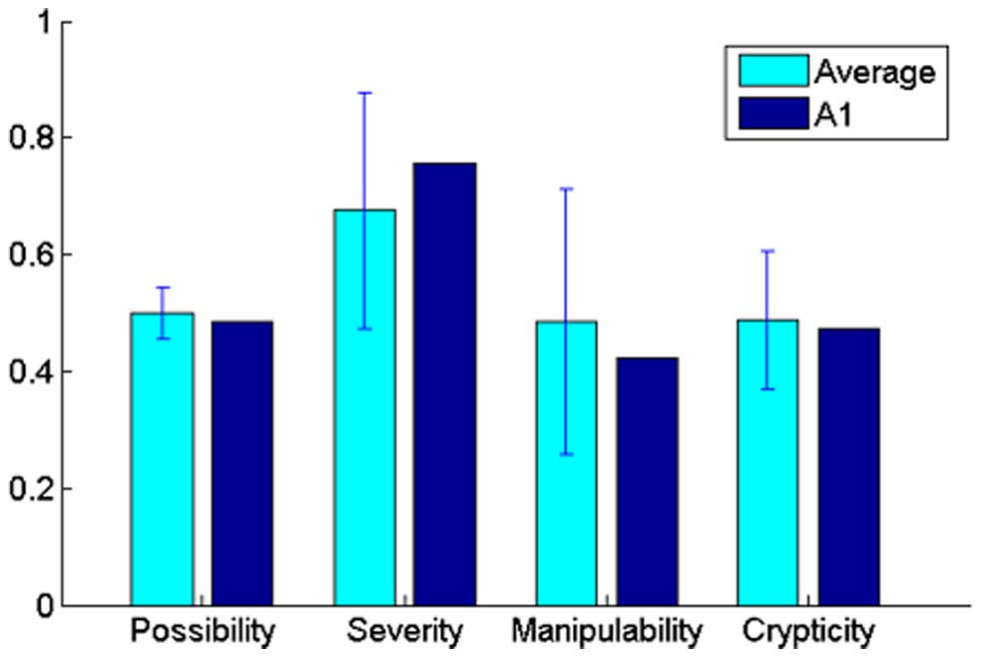
Quantitative values of A1 among browsers.

In order to observe the relative performance of A1, we selected 9 other web browsers and calculated the average and variance of the corresponding four metrics. The values of different metrics were further normalized into [0, 1] and compared with those of A1 in Fig. 9. It can be seen that the consequence of privacy leak behavior of A1 is more severe than the average consequence of other browsers, while its manipulability is lower than them. As for possibility and crypticity, A1 and other browers are at the same level.

In summary, this case study has well demonstrated that the developed approach is effectiveness in quatitatively analyzing the privacy leak behavior of software applications.

### Comparative Analysis on Different Software Categories

**Figure 10:**
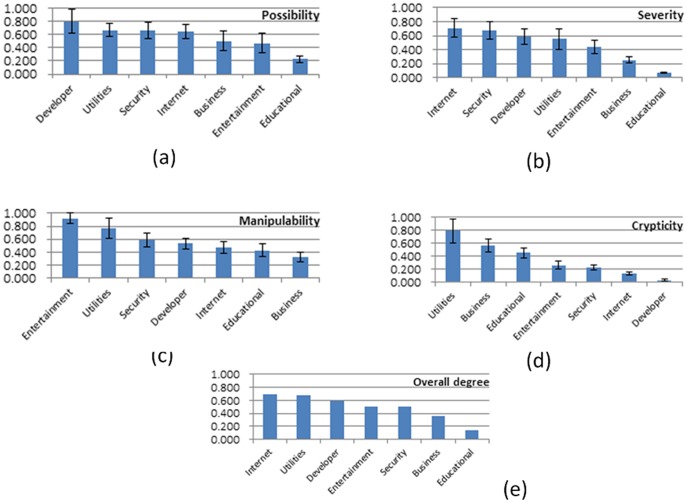
The four metrics and the overall degree of the seven categories of software applications.

In order to demonstrate the value and merits of the developed PPN based analysis approach, in this subsection we employ it to investigate the privacy leak behavior of different software categories. For each software category, we calculated the average and standard deviation of the four quantitative metrics and futher obtained the overall degree of privacy leak behaviors using the PLEAS algorithm. The results are presented in Table 2. Figure 10 presents the corresponding ordered result for each metric.

**Table 2:** quantitative metrics of different software categories.

Category	P		S		M		C		O
	*avg.*	*std.dev.*	*avg.*	*std.dev.*	*avg.*	*std.dev.*	*avg.*	*std.dev.*	
Entertainment	0.473	0.152	0.434	0.095	0.920	0.080	0.265	0.062	0.510
Utilities	0.669	0.102	0.555	0.148	0.534	0.081	0.792	0.185	0.677
Internet	0.649	0.105	0.709	0.130	0.473	0.087	0.135	0.026	0.695
Business	0.503	0.153	0.255	0.042	0.324	0.068	0.036	0.009	0.354
Educational	0.230	0.049	0.075	0.012	0.431	0.095	0.454	0.080	0.145
Developer	0.805	0.176	0.589	0.110	0.765	0.156	0.563	0.097	0.595
Security	0.666	0.121	0.674	0.126	0.588	0.107	0.231	0.035	0.506

Fig. 10 (a) shows that the category with the largest possibility is developer tools, because most developer tools not only manipulate many kinds of system resource such as file, processes and services, but also communicate with their remote servers such as code repository. The system utilities, security software and Internet software also have chances to access different kinds of privacy data and send them out via certain network operations, such as, updating database, getting new versions or communicating with service providers. Business software, entertainment software and educational software usually focus on local operations in their own data spaces and thus have fewer chances to form complete leak paths.

Fig. 10 (b) indicates that Internet software, security software and developer software are able to access most kinds of privacy data and have chances to send them out. Their behavior paths contain leak paths in all kinds of PPN modules such that the severity parameter 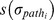 is usually larger than that of other software categories. Therefore, the severity of these categories is high. Due to similar reasons, entertainment software, business software and educational software have low severity.

High manipulability means that the software has more partial privacy leak paths. Fig. 10 (c) shows that entertainment software and utilities software have high manipulability because they usually collect some privacy data but do not send them out. Therefore, they often contain partial leak paths with only a source position, but without a discrimination position in the corresponding PPNs. Educational software and business software usually do not access privacy data. Consequently, their manipulability is low.

As shown in Fig.10 (d), obfuscating techniques involved in software design are often for the encryption of internal implementation and therefore immune to most of the reverse engineering tools. Software like utilities and business software which are not free usually contain many such techniques so that their crypticity is high. For developer tools that are usually open source, their crypticity is low.

As a comprehensive evaluation metric, the overall privacy leak degree of the seven software categories is presented in Fig.10 (e). It can be observed that Internet software, system utilizes and developer tools have a high privacy leak degree and educational software has the lowest degree. It should be pointed out that this is a general result for different software categories and it may not be appropriate for some special software tools. From this result, we can conclude that privacy leak behavior of software is mainly affected by three factors, namely, the chances that the application has to access important privacy data sources, the network function for transferring privacy data, and the crypticity for concealling the unauthorized operations.

Comparing the order of the seven software categories on different metrics, we find that the relationships between possibility and other three metrics are obviously verified. The possibility of Internet software, developer tools and system utility is high, and other three metrics of them are also higher than other software categories. The relation that severity and manipulability are complementary to each other is also verified. Internet software, security software, developer tools, utilities and entertainment software have reverse orders on severity and manipulability, respetively. The order of different software categories on crypticity implies the relationship we presented in the previous section. Although the other metrics of some software categories are low, they still have privacy leak threats if they have high crypticity. For example, the overall privacy leak degree of entertainment software is high, because its crypticity is high, although it has low possibility and severity.

From the above experimental results and reasonable explanations, we can conclude that the developed PPN based approach is an effective tool for quantitatively analyzeing privacy leak behavior of different software applications.

## Supporting Information

Supporting Information S1 Supporting Materials.
(DOCX)
