# Supplementary material for: Comprehensive Quantitative Analysis on Privacy Leak Behavior
Source: PLoS One. 2013 Sep 16;8(9):e73410. doi: 10.1371/journal.pone.0073410 (PMC3774722; doi:10.1371/journal.pone.0073410)
Supplement: Supporting Information S1 — Supporting Materials. (DOCX) [file pone.0073410.s001.docx]

# Supportting materials

## The algorithm of metrics calculation

Algorithm 1: The calculation of the four metrics

**Input**

*CS*: The call sequence of the software under test

**Output**

*P*: possibility; *S*: severity; *M*: manipulability; *C*: crypticity

**Begin**

/* Collect and filter the system calls of the tested software application.*/

**for** *c* **in** *CS*

/* if c does not satisfy the condition, drop it from *CS*. */

**if** **not** satisfy(*c*) **then**

drop(*c*, *CS*)

/* Trigger PPN *pn* with filtered system call sequence. */

**else**

trigger(c, pn)

/* insert the move path of token into *BPS* */

insert(*inst_c_*.*proc*, *BPS*)

/* Calculate the metrics by formula (1) to (4) */

[*P*, *S*, *M*, *C*] = [*P*(*BPS*), *S*(*BPS*), *M*(*BPS*), *C*(*BPS*)]

**End**

Algorithm 1 evaluates the system calls of the tested software application, and outputs the four quantitative metrics. First, we collect the system calls by monitoring the execution of the software on our experiment platform. Next, we check the collected system call sequence by two kinds of conditions. One is to check whether the call name is in the typical PPN modules. The other is to consider whether the parameters are related to our definition in the variable sets. Third, we trigger the PPN with the filtered system call sequence, perform the predefined operations. Fourth, the calls are processed, the moving trace of the tokens forms the BPS of the tested application. Finally, we calculate the four metrics based on the BPS.

## Quantification algorithm of overall privacy leak degree

The main steps involved in PLEAS are as follows.

Firstly, we put four metrics of different software into a matrix *M* as presented in Fig. 11, then normalize the data. For each element *m_ij_* in *M*, we calculate the normalized value $\tilde{m}_{ij}$ and vector $\tilde{x}_{i}$as in (5):

| $\tilde{m}_{ij}=\frac{m_{ij}-\mu_{ij}}{s_{i}}$ $\tilde{x}_{i}=\frac{x_{i}-\mu_{i}}{s_{i}}$, i = 1, 2, 3, 4 and j=1, 2, … , n | (5) |
| --- | --- |

Where $\mu_{ij}=\frac{1}{n}\sum_{j=1}^{n} a_{ij}$, $s_{i}=\sqrt{\frac{1}{n-1}\sum_{j=1}^{n} (m_{ij}-u_{ij})^{2}}$.

Secondly, we calculate the covariance matrix *R* = $r_{ij(4\times4)}$ from *M*:

| , i, j = 1, 2, 3, 4 | (6) |
| --- | --- |

from which the candidate principal component (PC) with the eigenvalue and eigenvector of R is calculated. Assuming that the eigenvalues are *λ_1_ ≥ λ_2_ ≥ λ_3_ ≥ λ_4_* ≥ 0, and the corresponding eigenvectors are *u_1_*, *u_2_*, *u_3_*, *u_4_*, where *u_i_* = [*u_1i_*, *u_2i_*, *u_3i_*, *u_4i_*]^T^, then the candidate principal components are:

|  | (7) |
| --- | --- |

Thirdly we select principal components from the candidates. The number of principal components is less than or equal to the number of original variables. This selection is from the information proportion *b_i_* of the *pc_i_*. The first principal component is the most informative, and each succeeding component in turn has a less variance possible. *b_i_* is calculated by eigenvalues:

| $b_{i}=\frac{\lambda_{i}}{\sum_{k=1}^{4} \lambda_{k}}$ | (8) |
| --- | --- |

and the cumulative information proportion *a_p_* is calculated based on *b_i_*:

| $a_{p}=\sum_{k=1}^{p} b_{k},b_{k}>b_{k+1}$ | (9) |
| --- | --- |

when *a_p_* approaches 1, we select *pc_1_*, *pc_2_* ... *pc_p_* as the principal components.

Finally we evaluate the values *X* of each alternative with the principal components selected in step 7 as in (10):

| $O=\sum_{k=1}^{p} \lambda_{k}\cdot pc_{k}$ | (10) |
| --- | --- |

*O* is the ranking value to evaluate the overall degree of privacy leak of the tested software application.The whole procedure is presented in Algorithm 2.

Algorithm 2: PLEAS

**Input**

*A_1_, A_2_,… ,A_n_*: metrics tuple of *n* tested software applications, where *A_i_* = (*p_i_*, *s_i_*, *m_i_*, *c_i_*).

**Output**

*O_1_, O_2_,… ,O_n_*: overall privacy leak degree from *A_1_* to *A_n_*

**Begin**

/* Normalize metrics data by formula (5). */

*M* = [*A_1_, A_2_,… ,A_n_*]^T^

*M’* = normalize(*M*)

/* Calculate the covariance matrix by formula (6). */

*R* = covariance(*M*)

/* Calculate the eigenvalue and eigenvector of R. */

*U* = [*u_1_*, *u_2_*, *u_3_*, *u_4_*] = eigenvector (*R*)

*Λ* = [*λ_1_, λ_2_, λ_3_, λ_4_*] = eigenvalue(*R*)

/* Select principal component by formula (7) to (9). */

**for** *i* = 1 **to** 4

*b_i_* = *b_i_* + *λ_i_*

*pc_i_* = *u_i_* ∙ [*x_1_*, *x_2_*, *x_3_*, *x_4_*]^T^

*p* = *i*

/* The proportion threshold of principal component is 80% */

**if** $\frac{b_{i}}{\sum_{i=1}^{4} \lambda_{i}}>0.8$ **then** **break**

/* Evaluate the alternatives by formula (10). */

**for** *i* = 1 **to** *n*

**for** *j* = 1 **to** *p*

*O_i_* = *O_i_* +*λ_i_* ∙ *pc_i_*

**End**

The PCs found by PLEAS can assign the optimal weights of different metrics without the subjective assumption to maximize the variance of the metric vector. Therefore, it can effectively evaluate the overall degree of privacy leak based on the four metrics. Proposition 1 is a formal proof of this characteristic.

**Proposition 1.** *Let X = [x_1_, x_2_, … , x_p_] be variable vector which denotes the metrics of privacy leak behavior, W = [w_1_, w_2_, … , w_p_] be the weight factor of each metric, λ_1_ ≥ λ_2_ ≥ … ≥ λ_p_ ≥ 0 be the eigenvalue of the variance matrix Var(X), the corresponding eigenvector is* *u_1_, u_2_, … , u_p_. We have$\mathop{\text{max}}\limits_{\text{W：}\left\|\text{W}\right\|=1} \text{Var}(W^{\text{T}}\text{X}),\text{iff} \text{W}=\mu_{1}$.*

**Proof:** Assume that M =$\mathop{\text{max}}\limits_{\text{W：}\left\|\text{W}\right\|=1} \text{Var}(W^{\text{T}}\text{X})=\mathop{\text{max}}\limits_{\text{W：}\left\|\text{W}\right\|=1} \text{W}^{\text{T}}\text{Var}(X)W$. Let$\Gamma\wedge\Gamma^{\text{T}}$be the Jordan Decomposition of Var(X), then $\Gamma$= (*u_1_*, *u_2_*, *…* , *u_p_*) $\Lambda$diag(*λ_1_*, *λ_2_*, *…*, *λ_p_*). Let *Y=$\text{W}^{\text{T}}\Gamma$.* Because Y^T^Y =$\text{W}^{\text{T}}\Gamma\Gamma^{\text{T}}\text{W}$ = W^T^W= 1, we have

M =$\mathop{\text{max}}\limits_{\text{W：}\left\|\text{W}\right\|=1} \text{W}^{\text{T}}\Gamma\wedge\Gamma^{\text{T}}\text{W}$

=$\mathop{\text{max}}\limits_{\text{Y：}\left\|\text{Y}\right\|=1} \text{Y}\wedge\text{T}^{\text{T}}$

=$\mathop{\text{max}}\limits_{\text{Y：}\left\|\text{Y}\right\|=1} \sum_{\text{i}=1}^{\text{p}} \lambda_{\text{i}}\text{Y}_{\text{i}}^{2}\leq\lambda_{1}\mathop{\text{max}}\limits_{\text{Y：}\left\|\text{Y}\right\|=1} \sum_{\text{i}=1}^{\text{p}} \text{Y}_{\text{i}}^{2}=\lambda_{1}$.

The maximum is obtained when *Y* = [1, 0,…,0]^T^, thus $W=u_{1}$ . Theorem 2 holds.

*W^T^X* is the first principal component selected based on Equation (7). Similarly, we can select other principals using Equations (8) and (9).
